# Gender Differences in Lower Limb Strength and Endurance Among Saudi Adolescents: A Cross-Sectional Study on the Limited Role of Body Mass Index

**DOI:** 10.3390/children12070899

**Published:** 2025-07-08

**Authors:** Asma Alonazi, Fay Alsunaid, Latifa Alofaisan, Mohammed Ghassan Alqarni, Jasem Alhumoud, Faizan Kashoo

**Affiliations:** 1 Department of Physical Therapy and Health Rehabilitation, College of Applied Medical Sciences, Majmaah University, Riyadh 11952, Saudi Arabia; f.kashoo@mu.edu.sa; 2 Tumair Hospital, Tumair 11943, Saudi Arabia; 3 Physical Therapy Department, Security Forces Hospital, Riyadh 12625, Saudi Arabia; 4 Department of Physical Therapy and Rehabilitation, Savan Medical Centre, Riyadh 14244, Saudi Arabia; 5 Department of Physical Therapy and Rehabilitation, Earthy Center, Riyadh 12481, Saudi Arabia

**Keywords:** adolescent health, body mass index, muscle strength, motor performance, physical activity, obesity, Saudi Arabia

## Abstract

**Background**: Understanding the relationship between physical fitness and body mass index (BMI) is critical for promoting adolescent health, particularly in Saudi Arabia, where cultural norms and rising obesity rates present unique challenges. This study aimed to investigate the impact of BMI, gender, and physical activity levels on lower limb strength and endurance, as measured by the Standing Long Jump (SLJ) and the 1 min Sit-to-Stand (STS) test, respectively. **Methods**: This cross-sectional study included 100 healthy Saudi adolescents (44 boys, 56 girls) aged 10–18 years. Lower limb strength and endurance were assessed using SLJ (cm) and STS (repetitions/min). Anthropometric measurements included BMI (kg/m^2^), weight (kg), and height (cm), while physical activity was assessed using the Physical Activity Questionnaire for Adolescents (PAQ-A). Mediation analysis was conducted to examine the potential indirect effects of BMI, PAQ-A score, and age on the relationship between SLJ and STS performance. **Results**: Boys significantly outperformed girls in both the STS (mean difference = 25.2 repetitions/min; *p* < 0.001) and SLJ (mean difference = 73.4 cm; *p* < 0.001). No significant gender differences were found in PAQ-A scores (*p* = 0.987). A strong positive correlation was observed between SLJ and STS performance (*r* = 0.768; *p* < 0.01). BMI was not significantly correlated with SLJ or STS performance. STS repetitions predicted superior SLJ performance both before (β = 0.55, *p* < 0.001) and after (β = 0.47, *p* = 0.004) adjustment for BMI, age, PAQ score, and gender. BMI transmitted only a small, non-significant share of this link (indirect β = 0.08, *p* = 0.122), indicating that the STS–SLJ association is largely direct (model R^2^ for SLJ = 0.84). **Conclusions**: Explosive lower limb strength and gender were significant predictors of lower-body endurance, whereas BMI showed a limited association with performance. These findings underscore the importance of incorporating gender-specific strategies in adolescent fitness assessments and interventions, with a cautionary interpretation of BMI as a performance indicator.

## 1. Introduction

The global prevalence of adolescent obesity has reached alarming levels, posing serious public health challenges [1,2]. In Saudi Arabia, this issue is particularly acute, with recent studies reporting that one in five children and 19.4% of adolescents are classified as overweight or obese [3,4,5,6]. The urgency of this issue is underscored by the fact that obesity significantly increases the risks of chronic conditions such as type 2 diabetes, cardiovascular disease, and orthopedic complications while placing a substantial burden on the healthcare system [5,7]. Moreover, overweight and obese Saudi children are likely to carry excess weight into adulthood [8], increasing their vulnerability to long-term health issues. These trends highlight the urgent need to understand how obesity impacts physical fitness and performance, particularly among Saudi adolescents. Assessing physical fitness in relation to body mass index (BMI) is crucial for early intervention and prevention efforts, as it guides targeted strategies to improve health outcomes [9].

Physical fitness is a multidimensional health construct that refers to an individual’s ability to carry out daily tasks with vigor and alertness, without undue fatigue, and with ample energy to enjoy leisure pursuits such as hiking, swimming, or playing sports and respond to emergencies like a sudden need for physical exertion or a life-threatening situation. It encompasses various components of health-related performance, including muscular strength, cardiovascular endurance, flexibility, speed, and body composition [10,11]. Physical performance, in contrast, refers to the observable ability to execute specific motor tasks effectively, such as jumping, standing, or walking, which reflect an individual’s functional capabilities [12]. Explosive strength, a subset of muscular strength, is defined as the ability to exert maximal force in the shortest time possible, typically used in dynamic activities such as jumping or sprinting [13]. While these constructs are related, muscular strength, for example, supports endurance during prolonged activities, while flexibility enhances functional movements, reducing the risk of injury. These distinctions have practical implications, as they help in evaluating adolescents’ functional abilities and identifying early indicators of health risks.

Lower-limb muscular strength and endurance are vital attributes that support the rapid physical, hormonal, and cognitive changes that occur in adolescence. Enhanced lower-body strength and endurance during this period are associated with improved mobility, physical independence, bone density, and long-term health outcomes [14]. This study utilizes two standardized, field-based assessments: the Standing Long Jump (SLJ), which evaluates explosive lower limb strength, and the 1 min Sit-to-Stand (STS) test, which assesses lower-body muscular endurance and functional mobility [15,16]. These tests are widely validated, easy to administer in school settings, and sensitive to developmental and fitness-related changes in youth populations [15,16]. Their use allows for practical screening of both power and endurance within the same participants.

While the benefits of physical activity (PA) in reducing health risks and enhancing quality of life are well-documented, maintaining muscular strength and endurance during adolescence is crucial. These attributes not only mitigate the adverse effects of obesity [17] but also significantly enhance the ability to perform daily activities such as walking, climbing stairs, and participating in sports. This empowerment in daily life is a direct result of maintaining muscular strength and endurance, which also contributes to bone health and reduces the risk of osteoporosis and obesity [18,19].

Adolescents with higher BMI values often demonstrate poorer performance in weight-bearing tasks than their normal-weight peers, regardless of sex, due to excess body mass that impairs functional movement and endurance [20]. Adolescents with body composition values within the normal range tend to demonstrate higher levels of physical fitness [21]. On the other hand, underweight adolescents may also display reduced strength and energy availability, highlighting the need to examine the full spectrum of BMI categories. Despite a growing body of international research, the scarcity of regional data examining how BMI categories affect functional fitness in Saudi youth is a significant gap that, once filled, could greatly inform and influence health policies for adolescents.

Gender differences, biological maturity, and hormonal status further complicate the relationship during adolescence. Males typically exhibit higher levels of strength and endurance, primarily due to greater muscle mass and hormonal influences [22]. In contrast, the influence of sociocultural norms on females is evident in their engagement in lower-intensity physical activity. Adolescents experience varied growth trajectories that influence muscle development and coordination [23]. However, the significant variability in biological maturity poses a major challenge for age-standardized comparisons, making the study of gender differences in adolescent development a complex and nuanced task [24].

Studies have shown that obesity has a negative impact on performance in weight-bearing tasks [25]. However, there is a pressing need for localized research in Saudi Arabia, specifically focusing on underweight and normal-weight adolescents. Their physical performance profiles may differ substantially from those of overweight or obese peers, and understanding these differences is crucial. This research has the potential to significantly impact our understanding of adolescent health and performance. Many studies overlook the complexities introduced by factors such as gender, age, and maturity status, further highlighting the urgency of this research. Existing literature often generalizes findings across all BMI groups, neglecting the nuanced interactions between weight status, physical activity, age, and gender. The absence of population-specific data further limits the applicability of global findings to Saudi local health policy and planning.

To address this gap, with a specific focus on Saudi adolescents, this study aims to examine the relationships between BMI, gender, physical activity levels (measured by the Physical Activity Questionnaire for Adolescents (PAQ-A), and lower-limb physical performance in the Saudi population. The findings of this study could significantly contribute to our understanding of how BMI and PAQ-A scores impact performance in the SLJ and STS tests, as well as whether explosive strength (measured in the SLJ) mediates the relationship between BMI/PAQ-A and endurance (assessed in the STS).

We hypothesize that:

(1) Higher BMI is associated with lower STS and SLJ performance;
(2) Boys will outperform girls in both SLJ and STS; and
(3) SLJ performance mediates the relationship between BMI, physical activity, and STS outcomes.

## 2. Materials and Methods

### 2.1. Study Design, Setting, and Participants

A cross-sectional observational study was conducted in the Al-Majmaah province of Riyadh, Saudi Arabia. Ethical approval for the research was obtained from the Institutional Review Board (IRB) of Majmaah University under project number MUREC-Nov.4/COM-2021/90-2. One hundred healthy adolescents aged 10 to 18 were recruited from schools across various cities, including Riyadh, Majmaah, Hawtat Sudair, and Tumair. Prior to data collection, we performed an a priori power analysis (G*Power 3.1) using a two-tailed test, α = 0.05, and power = 0.80. Assuming a medium correlation (r = 0.30) between standing long jump (SLJ) and sit-to-stand (STS) performance, the required sample was 84. We recruited 100 participants, achieving a power of 0.87. Participants were recruited using convenience sampling from selected schools. Adolescents with recent illnesses, surgeries, or musculoskeletal injuries were excluded.

### 2.2. Outcome Measures

#### 2.2.1. Anthropometrics Data

The research team collected data on participants’ age, gender, weight (kg), height (cm), and Body Mass Index (BMI) (kg/m^2^). SLJ and STS scores were initially normalized for height and weight to account for anthropometric differences. Specifically, SLJ distance was divided by body height (SLJ-H, cm·cm^−1^), and STS repetitions were divided by body mass (STS-W, reps·kg^−1^). These normalized scores were used in descriptive analyses to minimize confounding due to gender-related size differences. However, for inferential statistical tests, raw scores were retained to preserve variance associated with actual performance, particularly given the sample’s homogeneous BMI distribution. All statistical analyses used these normalized scores.

#### 2.2.2. Physical Activity Questionnaire (PAQ-A)

Participants completed a self-reported 7-day recall using the PAQ-A, a validated tool suitable for adolescents aged 10–18. This questionnaire was designed and developed by Kowalski K. et al. [26]. The PAQ-A produces a mean score ranging from 1 to 5, with higher scores indicating greater physical activity.

#### 2.2.3. Physical Performance Tests

One-Minute Sit-to-Stand (STS) Test: The research team utilized the 1 min STS test to assess quadriceps strength in relation to body weight. Each participant completed repeated cycles of sitting and standing for over one minute without using their arms for support [15,27].

Standing Long Jump (SLJ) Test: The research team used the SLJ test to measure lower limb strength and power. Participants were instructed to jump as far forward as possible from standing, with the best distance recorded from three attempts [28].

### 2.3. Study Procedure

Before data collection began, the research team, comprising physical therapists and trained researchers, visited several high schools to coordinate the recruitment process. Informed consent forms detailing the study’s purpose, procedures, benefits, and risks were distributed to parents through school principals and also sent via email. Private interviews were conducted with students to confirm their voluntary participation and to provide them with the opportunity to ask questions. Assent was formally obtained from students during these school visits. Researchers emphasized participants’ right to withdraw at any time without penalty. Once approvals were secured, the researcher recorded sociodemographic information, including age, gender, weight, and height. Anthropometric measurements were taken by a single trained researcher using a Tanita BC-545N digital scale (Tokyo, Japan) (for weight) and a Seca 213 stadiometer (Hamburg, Germany) (for height).

In contrast, a second researcher reviewed the data for consistency. Participants were instructed to remove their shoes and heavy clothing and to refrain from eating or drinking for at least two hours prior to measurement. For weight, participants stood upright, feet flat and evenly spaced on the calibrated digital scale. For height measurement, they stood barefoot, with their heels together and their bodies aligned against the stadiometer, their heads positioned in the Frankfort horizontal plane. Height and weight were recorded to the nearest 0.1 cm and 0.1 kg, respectively. Body Mass Index (BMI) was calculated using the formula: BMI = weight (kg)/height (m)^2^. All measurements were taken in a temperature-controlled setting to ensure accuracy and reliability.

After the anthropometric assessment, participants completed the Physical Activity Questionnaire for Adolescents (PAQ-A), which evaluates self-reported physical activity over the past seven days. Instructions were provided verbally and in writing; researchers assisted younger participants as needed to ensure comprehension and accurate responses. The PAQ-A was scored on a 5-point scale, with higher scores indicating greater activity levels. The completed questionnaires are then reviewed for completeness, and the data are scored according to PAQ-A guidelines to derive an overall physical activity score. This process ensures an accurate and reliable assessment of adolescents’ physical activity levels.

For physical performance assessments, the 1 min Sit-to-Stand (STS) test was conducted using the IKEA SMEUR LLEN swivel chair (Model 005.034.35), which offers an adjustable seat height (40–52 cm) to ensure each participant’s knees were at a 90-degree angle. The chair’s seat dimensions (40 cm width × 39 cm depth) and non-slip base provided a stable, armrest-free platform, preventing the need for arm assistance during the test. Higher repetition counts indicated greater lower-body strength and endurance.

The Standing Long Jump (SLJ) test was performed on a hard floor. Participants stood with their feet parallel at the start line and jumped forward using arm propulsion, landing on both feet. Three attempts were allowed, and the best distance, measured from the start line to the nearest point of landing, was recorded in centimeters. A standardized three-minute rest interval was given to the participants between attempts to prevent fatigue. All test procedures were conducted under direct supervision to ensure safety, consistency, and standardized data collection.

### 2.4. Statistical Analysis

Univariate analyses were conducted to examine the relationship between demographic variables and the study outcomes, with demographic data used as covariates. Descriptive statistics, including means and standard deviations, were calculated for continuous variables (e.g., age, height, weight, and BMI). For qualitative variables such as BMI category, frequency distributions were examined, presenting the number and percentage of participants in each group. Analysis of covariance (ANCOVA) was conducted to assess differences in physical performance outcomes (STS and SLJ) across gender and BMI categories, with adjustments for relevant covariates. The Bonferroni method was used to control for Type I errors in multiple comparisons.

The model specification was theory-driven and informed by zero-order correlations. In physiology, BMI, habitual activity level (as measured by the PAQ), and biological maturation (as indicated by age) are all considered potential pathways linking neuromuscular endurance to explosive power. We fitted a single-mediation model that treated SLJ as the predictor, STS as the outcome, and BMI as the mediator, controlling for age, PAQ, and gender. The model was estimated in JASP v0.18.1 using maximum-likelihood, with indirect effects evaluated via a bias-corrected 5000-sample bootstrap; delta-method standard errors and 95% confidence intervals are reported, and an indirect pathway was regarded as significant when its CI excluded zero (α = 0.05).

## 3. Results

The demographic table reveals significant differences between boys and girls in height and weight (*p* < 0.001). Boys exhibited significantly greater height and weight compared to girls. However, no significant differences were observed in age or BMI between the two groups (*p* > 0.05). Furthermore, the distribution of BMI categories did not differ significantly between boys and girls (*p* = 0.314) (Table 1).

Our study has revealed a strong positive correlation between STS and SLJ performances (r = 0.768, *p* < 0.01), indicating a significant association between these two measures of lower-body strength and power. Additionally, significant positive correlations were found between STS and height (r = 0.506, *p* < 0.01) as well as between SLJ and height (r = 0.650, *p* < 0.01), indicating that taller participants tend to perform better in both tests (Table 2).

BMI did not show a statistically significant correlation with either STS or SLJ performance (*p* < 0.05). However, BMI exhibited a strong positive correlation with weight (r = 0.861, *p* < 0.01) and a moderate positive correlation with height (r = 0.171, *p* < 0.01), underscoring the association between body weight and height in adolescents.

### 3.1. Gender Disparities for Sit-to-Stand Test, Standing Long Jump, and Physical Activity

Analysis of Covariance was used to evaluate STS test scores as the dependent variable with demographic variables as covariates; a significant difference was found between genders. Boys exhibited a noteworthy mean difference of 25.238 (SE = 3.104, *p* < 0.001, 95% CI [19.076, 31.400]) compared to girls. Similarly, for SLJ, a substantial disparity emerged, with a significant mean difference of 73.449 (SE = 5.434, *p* < 0.001, 95% CI [62.660, 84.238]). Conversely, PAQ did not show a significant difference, with a mean difference of −0.061 (SE = 3.711, *p* = 0.987, 95% CI [−7.430, 7.307]). These findings highlight the influence of gender on STS and SLJ outcomes while indicating no significant impact on PAQ scores.

### 3.2. Comparison of Sit-to-Stand, Standing Long Jump, and Physical Activity Across BMI Categories

Analysis of Covariance was used to evaluate physical performance differences across BMI categories in adolescents, with adjustments made for covariates and Type I error controlled using the Bonferroni method. The results revealed no significant differences in Sit-to-Stand (STS) performance among Underweight, Healthy Weight, Overweight, and Obese individuals (*p* > 0.05). Similarly, no significant differences were found in Standing Long Jump (SLJ) performance across BMI categories (*p* > 0.05). Additionally, there were no significant differences in Physical Activity Questionnaire (PAQ) scores across BMI categories after adjusting for covariates (*p* > 0.05).

### 3.3. Mediation Analysis

**Figure 1:**
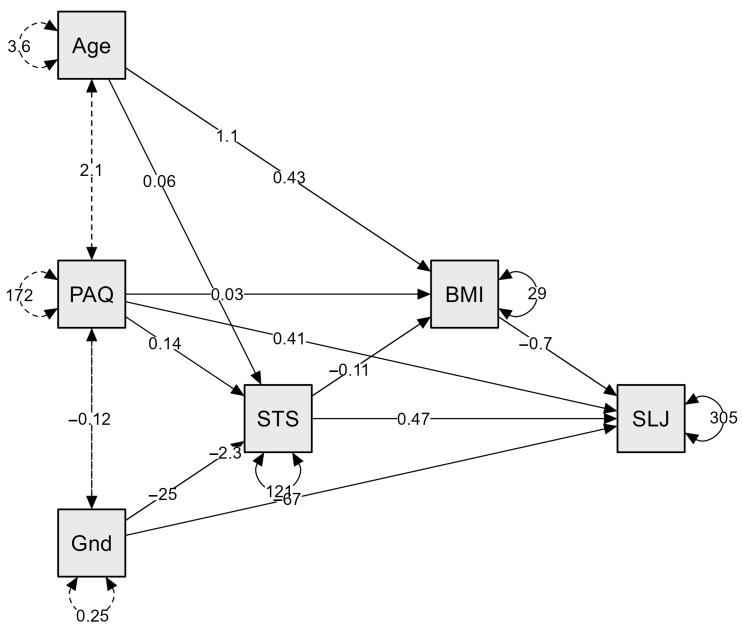
BMI-mediated path model linking sit-to-stand repetitions (STS) to standing long-jump distance (SLJ), with age, PAQ score, and gender entered as covariates. Note: Path diagram for the single-mediator model testing whether body mass index (BMI) transmits the effect of one-minute sit-to-stand repetitions (STS) to standing long-jump distance (SLJ). Solid arrows display unstandardized maximum-likelihood path coefficients; dashed, double-headed arrows mark correlations among exogenous covariates (Age, PAQ = Physical Activity Questionnaire score, and Gnd = gender). Curved arrows attached to each endogenous box represent the residual variances (numerical values adjacent to the arcs). All coefficients are accompanied by their point estimates (two-tail *p* < 0.05 paths are significant in the accompanying text). For visual clarity, the thickness of the arrows is uniform; statistical significance is not graphically coded.

In the single-mediator model (using maximum likelihood estimation), we examined whether BMI mediates the effect of 1 min STS performance on SLJ, controlling for age, PAQ score, and gender. STS showed a significant positive association with SLJ both as a total effect, *b* = 0.55, *SE* = 0.16, *z* = 3.37, *p* < 0.001, 95% CI [0.27, 0.84] and as a direct effect after accounting for BMI, *b* = 0.47, *SE* = 0.16, *z* = 2.89, *p* = 0.004, 95% CI [0.18, 0.77]. Although STS predicted lower BMI (*b* = −0.11, *SE* = 0.05, *z* = −2.24, *p* = 0.025), and higher BMI predicted shorter SLJ distances (*b* = −0.70, *SE* = 0.33, *z* = −2.14, *p* = 0.033), the resulting indirect effect of STS on SLJ through BMI was small and non-significant, *b* = 0.08, *SE* = 0.05, *z* = 1.55, *p* = 0.122, 95% CI [0.01, 0.22]. Thus, in this cohort, the association between functional strength (STS) and explosive power (SLJ) appears to be predominantly direct. The final model reported 84% of the variance in SLJ (*R*^2^ = 0.84) but only 16% in BMI (*R*^2^ = 0.16), suggesting that additional determinants of adiposity were not captured in the analysis. (Table 3, Figure 1).

## 4. Discussion

This study investigated the influence of gender, body mass index (BMI), and physical activity levels on lower-body strength and endurance in Saudi adolescents, using the Sit-to-Stand (STS) and Standing Long Jump (SLJ) tests as performance measures. The results revealed significant gender-based differences, consistent with findings in the existing literature. Boys significantly outperformed girls in both the STS and SLJ tests, underscoring persistent gender disparities in adolescent physical performance [29,30,31,32]. These disparities are primarily attributed to physiological factors, including differences in muscle mass, longer limb length, and the influence of sex hormones that support strength development during puberty [29,30,31,32]. On average, boys completed 25.2 more STS repetitions and jumped 73.4 cm farther than girls, reflecting the cumulative effect of these biological advantages.

In examining gender and anthropometric differences in physical performance, our findings suggest that while boys tend to be taller and heavier than girls, there are no significant differences in age or BMI distribution between the two genders. Height has emerged as a contributing factor in both STS and SLJ performance, reinforcing the evidence that taller adolescents benefit from biomechanical advantages such as increased leverage and stride length [33,34]. Interestingly, BMI showed no significant correlation with either STS or SLJ performance, suggesting that BMI may not be directly associated with lower-body muscular strength or power in this population. However, given the cross-sectional design, these associations do not imply causality and should be interpreted with caution. A majority of participants were classified as having a healthy weight, with relatively few in the underweight or obese categories. This imbalance may have reduced the statistical power to detect meaningful differences in physical performance associated with extreme BMI levels. As such, these non-significant results should not be generalized to all BMI groups without further investigation using more balanced samples.

While overweight and obese individuals often exhibit poorer fitness outcomes, BMI may mask meaningful differences in strength and functional ability among adolescents with similar weight statuses [35,36,37]. These findings are consistent with prior research suggesting that BMI alone may be insufficient for predicting physical performance, particularly in non-obese adolescents [38,39,40,41]. Several studies have demonstrated that while BMI sometimes correlates with fitness, fat mass is a more accurate predictor of strength and endurance, highlighting BMI’s inability to differentiate between fat and lean muscle mass [42,43,44]. The absence of a significant relationship between BMI and physical test performance in our sample highlights the limitations of BMI in capturing variations in body composition, underscoring the need for a critical and urgent reevaluation of its use in sports science and health [38,39,40,41].

The mediation analysis further emphasizes the dominant roles of SLJ performance and gender in predicting STS outcomes, with BMI, age, and physical activity (as measured by the PAQ-A score) showing limited mediating effects. The direct relationship between SLJ and STS reflects the integrated nature of lower-body strength and functional endurance. While SLJ is an explosive movement and STS is an endurance-based test, their shared dependence on quadriceps strength may explain this linkage [45,46]. While BMI did not significantly mediate the relationship between SLJ and STS performance in this study, it remains a clinically relevant and widely used screening tool for identifying general weight status and associated health risks in youth populations. Its utility lies in its simplicity, accessibility, and cost-effectiveness, even if it may not fully capture the complexity of functional fitness performance.

While prior studies have suggested a strong influence of BMI and physical activity on adolescent fitness [20,33,38], our findings did not support mediation by either factor. For example, research using IPAQ in healthy young adults demonstrated that higher self-reported activity levels were associated with better performance on the 30-s and 1 min Sit-to-Stand tests, indicating that habitual activity enhances muscular endurance [47]. However, our findings did not reveal a significant relationship; the limited variability in PAQ-A scores among participants may have influenced this result. This discrepancy may stem from the limited variability in PAQ-A scores and the age appropriateness of the tool. These factors may have reduced our ability to detect subtle associations, particularly in a non-obese, moderately active sample. Regardless, this study opens up new avenues for future research to explore these subtle associations and their potential impact on adolescent fitness, underscoring the significance of our research in this field.

Our model demonstrated a substantial direct effect of SLJ on STS performance (β = 0.471, *p* < 0.01), with no significant indirect effects via BMI or PAQ-A. These results support the view that physiological differences (e.g., muscle mass and hormonal factors) are more predictive of endurance than anthropometric indices or activity measures. We acknowledge that our parallel mediation model was intentionally simple and did not include potentially important mediators, such as fat mass, lean body mass, pubertal stage, or nutritional factors. Future studies could incorporate these factors to provide a more complete picture.

Regression models supported these findings. The SLJ demonstrated its predictive power by explaining a substantial 86.4% of the variance while controlling for gender, age, and PAQ score; BMI had a modest adverse effect. In contrast, the STS model explained 56.3% of the variance, with SLJ being the only significant predictor, highlighting the potential utility of SLJ as a proxy measure for overall lower limb function.

The more substantial predictive value of gender and SLJ for STS performance highlights the need for nuanced fitness evaluations that extend beyond BMI, as well as strength-focused interventions targeting females’ adolescent physical performance to close performance gaps and promote equitable health outcomes.

### 4.1. Limitations

Despite providing valuable insights, this study has several limitations that underscore the need for further research. The relatively small sample size and non-random sampling, which was due to practical constraints, limit the generalizability of the findings. Participants were exclusively from the Riyadh region, which may not represent adolescents in other parts of Saudi Arabia. Additionally, the study did not control for important confounding variables such as sleep patterns, dietary habits, mental health, or hormonal levels, all of which may influence physical fitness outcomes. Most participants were of a healthy weight, which reduced the statistical power to detect performance differences across BMI categories. The underrepresentation of underweight and obese adolescents limits the generalizability of findings related to BMI. As a result, the non-significant associations between BMI and functional performance outcomes may reflect sampling limitations rather than the absence of actual effects. Future research should include larger and more evenly distributed samples across BMI classifications to improve subgroup comparisons and inference.

Furthermore, it is important to note that although normalization of SLJ and STS scores for height and weight was conducted descriptively to account for gender and body size differences, this adjustment was not retained in the final inferential analyses. This decision was made to preserve the variance attributable to actual motor output and performance, particularly given the limited variability in BMI across our sample. However, we acknowledge that omitting normalized scores in statistical testing may have introduced bias, especially in light of the observed gender differences in anthropometrics. Additionally, the wide age range (10 to 18 years) introduces developmental variability, yet biological maturity indicators, such as peak height velocity, were not assessed. Stratifying participants by maturity status or including developmental markers would enhance precision in future research.

Moreover, the use of the PAQ-A questionnaire poses significant challenges, particularly for children under 14, which could seriously affect the reliability and clarity of self-reported physical activity levels [26]. Although the IPAQ is widely used and has demonstrated acceptable validity in various populations, it remains subject to recall bias and social desirability bias, particularly among adolescents. Objective measurement tools, such as accelerometers or smartwatches, provide more accurate and reliable assessments of physical activity by capturing real-time data on step count, energy expenditure, and heart rate. Therefore, future studies are strongly encouraged to adopt such objective tools to improve the precision and validity of physical activity measurement in this population.

Although our power analysis indicated an adequate sample size for the primary outcomes, the limited number of participants in the extreme BMI categories may still have reduced sensitivity to detect BMI-related effects. It is essential to acknowledge that by focusing solely on lower limb strength and endurance, the study may have overlooked other crucial components of physical fitness, including aerobic capacity, flexibility, and overall health status. Including such measures would have provided a more comprehensive assessment of adolescents’ physical fitness, highlighting the need for a holistic approach in future research.

### 4.2. Recommendations for Future Research

Future studies should expand the sample size, ensure diverse participant recruitment, and consider additional variables, such as sleep patterns and dietary habits, to further enhance the understanding of this phenomenon. However, it is equally important to establish normative values for the SLJ test in Saudi populations. This benchmark for comparison enhances the applicability of the findings. Moreover, it is crucial to underscore the urgent need for further research to explore and address gender disparities in adolescent physical performance.

## 5. Conclusions

This study suggests that gender and SLJ performance are primary predictors of STS outcomes among Saudi adolescents. BMI alone does not adequately explain performance disparities in this sample, but remains a valuable general screening tool. Given the uneven distribution across BMI categories and the use of BMI as a proxy for body composition, these findings should be interpreted with caution. Intervention programs should emphasize strength-based assessments and training, particularly for female adolescents, to promote resilience and overall well-being. Future studies should include more diverse samples and direct measures of body composition to clarify the role of BMI in physical performance.

## Figures and Tables

**Table 1:** Demographic characteristics of the participants stratified by gender.

Variable	Boys (n = 44)	Girls (n = 56)	*p* Value
Age (years)	15.20 (0.90)	14.70 (2.40)	0.149 ^A^
Height (cm)	171.63 (11.08)	154.04 (8.41)	0.001 ^A^
Weight (kg)	68.58 (19.42)	54.68 (16.64)	0.001 ^A^
BMI (kg/m^2^)	22.99 (5.07)	22.87 (6.51)	0.920 ^A^
BMI category			
Underweight (%)	2 (4.5%)	4 (7.1%)	0.979 ^B^
Healthy weight (%)	26 (59.1%)	38 (67.9%)	0.590 ^B^
Overweight (%)	10 (22.7%)	5 (8.9%)	0.093 ^B^
Obese (%)	6 (13.6%)	9 (16.1%)	0.363 ^B^

Note: The data were analyzed using the student’s *t*-test (denoted as superscripted ^A^) for continuous variables (age, height, weight, and BMI) and the Chi-square test (denoted as superscripted ^B^) for categorical variables (BMI category).

**Table 2:** The Pearson’s correlation coefficients among tests and demographic variables.

Variable	STS	SLJ	PAQ	BMI	Age	Weight
SLJ	0.768 **					
PAQ	0.122	0.153				
BMI	−0.120	−0.093	0.058			
Age	0.114	0.118	0.084	0.341 **		
Weight	0.150	0.257 **	0.052	0.861 **	0.387 **	
Height	0.506 **	0.650 **	0.083	0.171	0.340 **	0.623 **

Note. STS = Sit-to-Stand; SLJ = Standing Long Jump; PAQ = Physical Activity Questionnaire; BMI = Body Mass Index; ** *p* < 0.01 (2-tailed).

**Table 3:** Coefficient table for regression modelling.

Path (Predictor ➔ Outcome)	*b*	SE	*z*	*p*	95% CI Lower	95% CI Upper
Direct & mediating paths						
STS ➔ SLJ	0.471	0.163	2.892	0.004	0.175	0.773
BMI ➔ SLJ	−0.696	0.325	−2.138	0.033	−1.477	−0.102
STS ➔ BMI	−0.109	0.049	−2.244	0.025	−0.205	−0.011
Covariate paths (controls)						
Age ➔ STS	0.057	0.59	0.096	0.923	−0.713	1.01
PAQ ➔ STS	0.138	0.084	1.638	0.101	−0.054	0.319
Gender ➔ STS	−24.811	2.238	−11.088	<0.001	−29.801	−20.313
Age ➔ BMI	1.072	0.288	3.727	<0.001	0.471	1.68
PAQ ➔ BMI	0.028	0.042	0.679	0.497	−0.064	0.123
Gender ➔ BMI	−2.288	1.63	−1.404	0.16	−6.141	1.035
Age ➔ SLJ	0.426	0.999	0.427	0.67	−1.442	2.651
PAQ ➔ SLJ	0.408	0.136	3.008	0.003	0.12	0.693
Gender ➔ SLJ	−67.226	5.356	−12.551	<0.001	−78.630	−55.862

Note. *b* = unstandardized coefficient; SE = standard error; CI = bias-corrected bootstrap 95% confidence interval (5000 resamples). STS = one-minute sit-to-stand repetitions; SLJ = standing long-jump distance (cm); BMI = body-mass index (kg·m^−2^); PAQ = Physical Activity Questionnaire score; Model R^2^: SLJ = 0.843, BMI = 0.161, STS = 0.563. The arrow symbol (➔) indicates the direction of the regression path, where the variable before the arrow is the predictor (independent variable) and the variable after the arrow is the outcome (dependent variable).

## Data Availability

Data are available upon request.
